# Abiotic and Biotic Stressors Causing Equivalent Mortality Induce Highly Variable Transcriptional Responses in the Soybean Aphid

**DOI:** 10.1534/g3.114.015149

**Published:** 2014-12-23

**Authors:** Laramy S. Enders, Ryan D. Bickel, Jennifer A. Brisson, Tiffany M. Heng-Moss, Blair D. Siegfried, Anthony J. Zera, Nicholas J. Miller

**Affiliations:** * Department of Entomology, University of Nebraska-Lincoln, Lincoln, Nebraska 68583-0816; † Department of Biology, University of Rochester, Rochester, New York 14627-0211; ‡ School of Biological Sciences, University of Nebraska-Lincoln, Lincoln, Nebraska 68583-0118

**Keywords:** transcriptomics, RNA-seq, heat, starvation, plant defense

## Abstract

Environmental stress affects basic organismal functioning and can cause physiological, developmental, and reproductive impairment. However, in many nonmodel organisms, the core molecular stress response remains poorly characterized and the extent to which stress-induced transcriptional changes differ across qualitatively different stress types is largely unexplored. The current study examines the molecular stress response of the soybean aphid (*Aphis glycines*) using RNA sequencing and compares transcriptional responses to multiple stressors (heat, starvation, and plant defenses) at a standardized stress level (27% adult mortality). Stress-induced transcriptional changes showed remarkable variation, with starvation, heat, and plant defensive stress altering the expression of 3985, 510, and 12 genes, respectively. Molecular responses showed little overlap across all three stressors. However, a common transcriptional stress response was identified under heat and starvation, involved with up-regulation of glycogen biosynthesis and molecular chaperones and down-regulation of bacterial endosymbiont cellular and insect cuticular components. Stressor-specific responses indicated heat affected expression of heat shock proteins and cuticular components, whereas starvation altered a diverse set of genes involved in primary metabolism, oxidative reductive processes, nucleosome and histone assembly, and the regulation of DNA repair and replication. Exposure to host plant defenses elicited the weakest response, of which half of the genes were of unknown function. This study highlights the need for standardizing stress levels when comparing across stress types and provides a basis for understanding the role of general *vs.* stressor specific molecular responses in aphids.

Stress is widespread in nature, driving ecological interactions and influencing the evolutionary trajectory of many organisms (Hoffmann and Hercus 2000; Frankham 2005; Steinberg 2012). There are numerous forms of stress, including extreme temperature, drought, pathogens, parasites, and even internal genetic stress caused by the expression of deleterious alleles (Hoffmann and Hercus 2000; Frankham 2005; Kristensen *et al.* 2005). It is well established that stress can elicit responses across broad categories of biological organization and a wide range of taxa (Bijlsma and Loeschcke 1997; Kassahn *et al.* 2009; Korsloot *et al.* 2010). However, because research into stress has progressed independently across several fields of biology, a general framework linking multiple aspects of stress response is currently lacking and limits our understanding of how organisms cope with environmental challenge (Schulte 2014). Approaches are needed that begin to unravel the molecular responses produced by exposure to different forms of stress and that make connections to observed effects on organismal fitness.

Organisms often are challenged simultaneously by multiple environmental stresses in their natural environment. The extent to which general *vs.* stressor-specific cellular responses exist has therefore been of long-standing interest in ecology and evolutionary biology (Hoffmann and Parsons 1991; López-Maury *et al.* 2008; Kassahn *et al.* 2009). It has been hypothesized that organisms have evolved coordinated networks of genes and pathways that respond to a variety of stressors, collectively considered the stress “defensome” (Steinberg 2012), which promote cross-protection and adaption in variable environments (López-Maury *et al.* 2008; Rangel 2011). The regulation of a core set of genes under a variety of stressors has been demonstrated in bacteria (Battesti *et al.* 2011), yeast (Gasch *et al.* 2000; Chen *et al.* 2003), plants (Ahuja *et al.* 2010), and animals (Kassahn *et al.* 2009; Korsloot *et al.* 2010). Additional research supporting the defensome hypothesis indicates a common set of stress responsive proteins exist as well (Kültz 2005; Wang *et al.* 2009; Tomanek 2011). However, organisms also may require fine-tuned stressor specific responses to adapt to changing environments. There is continued debate over the molecular basis of stress specificity, which may be achieved through stress-specific interactions with components of a general defensome, posttranslational modifications, and/or the compartmentalization of stress proteins (Kültz 2005).

Plant-insect systems provide an opportunity to investigate the role of the molecular stress defensome *vs.* stressor-specific responses in promoting adaptation under variable environments. Both partners in these systems suffer direct effects from abiotic stressors such as extreme temperature, but each also serves as a source of biotic stress for the other. The molecular basis of response to stress in plants has been intensively studied (see review by Ahuja *et al.* 2010); however, insect herbivores commonly are treated as a source of biotic stress for plants rather than active participants in the interaction (Bilgin *et al.* 2010). As a result, insect stress responses within plant-insect systems are less well characterized, particularly at the molecular level.

This study aims to characterize the molecular stress response of the soybean aphid (*Aphis glycines*). *Aphis glycines* is a cyclically parthenogenetic species that specializes on soybean (*Glycine max*) as a host plant. The species is native to east Asia and has become a major agricultural pest in North America since being introduced around 2000 (Hill *et al.* 2012). Our goal was to measure aphid transcriptional responses to two abiotic stressors (heat and starvation) and host plant imposed biotic stress. Specifically, we investigated the following questions: 1) Do aphids exhibit stress induced transcriptional patterns consistent with the stress defensome hypothesis and 2) to what extent does the magnitude of response elicited at the transcriptional level and specific components of the molecular response vary across different stress types? We define stress as causing a significant reduction in organismal fitness relative to benign conditions (Hoffmann and Parsons 1991). We standardized the effect of stress with respect to adult aphid mortality in order to compare molecular responses across qualitatively different stress types. Importantly, because aphids reproduce parthenogenetically, we were able to use a single aphid genotype, such that confounding effects of genetic variation among comparisons between stress types did not influence results.

## Materials and Methods

### Aphid rearing and stress treatments

In July 2011 a colony of soybean aphids (*A. glycines*) was established from a single viviparous parthenogenetic female collected in Madison, Wisconsin. Microsatellite markers developed by Kim *et al.* (2010) were used to confirm this clonal colony consisted of a single aphid genotype. Aphids were maintained continuously on a single soybean plant (variety KS4202) grown in a plastic Cone-tainer (Ray Leach Cone-tainer, Hummert International, Earth City, MO) and covered by a custom fitted cylindrical plastic cage (30.5 cm × 4.4 cm). Soybean variety KS4202 was used for aphid colony maintenance because it has not been demonstrated to adversely affect aphid survival or development (Pierson *et al.* 2010; Enders *et al.* 2014). Soybean plants used for aphid colony maintenance and experiments were grown in a greenhouse (16-hr light:8-hr dark photoperiod). The aphid colony was maintained in a growth chamber at 24 ± 1° and using a (16-hr light:8-hr dark photoperiod.

Age-synchronized adult aphids were exposed to the following treatments for 36 hr: 1) heat stress (34 ± 1°); 2) starvation stress; 3) plant defensive stress (aphid-resistant soybean); and 4) benign controls conditions (24 ± 1°, aphid-susceptible soybean). Groups of 20 apterous adult aphids were placed on a single soybean trifoliate (V1 vegetative stage) using a custom built plastic Petri-dish cage (8.9 cm × 2.5 cm) (Enders *et al.* 2014). For both the control and heat treatments, an aphid-tolerant soybean variety was used (KS4202) and for the plant stress treatment, an aphid-resistant soybean variety (PI243540) was used that expresses the resistance gene *Rag2* (Resistance to *Aphis glycines*). For the starvation treatment aphids were placed in small Petri dish cages (35 mm × 10 mm) with a mesh panel in the lid to allow for air circulation. Three experimental blocks were set up, each consisting of 12 replicate groups of 20 aphids per treatment. After 36 hr of exposure to the aforementioned four treatments, the total number of surviving adults and offspring produced were recorded. Surviving adults were then flash frozen and stored at −80°. Aphid material harvested from Block I of the experiment was used for transcriptomic analysis (RNA sequencing; RNA-seq) and Block III for reverse transcription quantitative polymerase chain reaction (RT-qPCR) validation of RNA-seq expression levels.

Stress intensity or stress level can be quantified by measuring the relative reduction in a measure of interest (*e.g.*, fitness) under stressful and benign conditions [*i.e.*, 1 − (Stress/Benign)], such that zero would be no stress and a score of 1 would be the maximum amount of stress (Fox and Reed 2011; Enders *et al.* 2014). We used aphid mortality to measure stress level. Plant defensive stress caused by *Rag2* could not be easily modulated; therefore, to achieve a standardized stress level, we adjusted the length of exposure to heat and starvation. Preliminary experiments determined all three stressors caused an equivalent increase in mortality relative to control conditions at 36 hr. Our preliminary data and previous work demonstrating cessation of reproduction in starved aphids (Ward and Dixon 1982; Kouamé and Mackauer 1992) and the temperature dependence of insect developmental rates (Ratte 1985) suggests aphid reproduction is highly variable under different stresses. Nymph production was therefore considered an unreliable measure of stress, and mortality was determined a better indicator. Survival and nymph production were analyzed by analysis of variance using the following fixed effects model: Environment (Control, Heat, Starvation, Plant Defense), Block (I, II, III) and Environment × Block. *Post hoc* multiple comparisons across the four environments were performed using Tukey HSD tests and *P* values adjusted for multiple comparisons. Under starvation stress, aphids did not produce offspring in the 36-hr period of measurement, and this treatment was not included in the analysis of nymph production. Raw fitness data are available in Supporting Information, Table S1.

### Transcriptomic methods and analysis

Total RNA was isolated and purified from groups of 32 whole adult aphids using the QIAGEN RNeasy extraction kit according to manufacturer protocols. Three RNA samples were prepared for each of the four experimental treatments (three stresses and control) by randomly pooling aphids from across the 12 experimental replicates. RNA integrity was confirmed using the Agilent 2100 Bioanalyzer, and RNA-seq was performed on the Illumina HiSequation 2000 platform at the University of Nebraska Medical Center Genomics Core facility. Sequencing resulted in 23.5 million total single-end (50 base pair) reads on average per biological replicate. Adapter sequences and low quality sequences were removed prior to further bioinformatic analysis. All raw reads were deposited in the Sequence Read Archive at National Center for Biotechnology Information under the accession number SRP050997.

Gene expression was estimated by mapping reads using Bowtie 1.0 (Langmead *et al.* 2009) to the whole adult *A. glycines* transcriptome assembled by Liu *et al.* (2012). On average, 56% of the total reads mapped to the transcriptome across replicates and treatments. An updated annotation of the *A. glycines* transcriptome was performed using the BLAST2GO platform (Conesa *et al.* 2005), which involved searching contigs against the GenBank nonredundant database using BLASTx algorithms and implementing Gene Ontology (GO) annotation using the Swiss-Prot database and InterProScan. Analysis of differential gene expression was performed in the R statistical environment (R Core Team 2012) using the program DESeq (Anders and Huber 2010) with a false discovery rate of 0.10. Stress-responsive genes were identified by comparing gene expression in the control treatment to each of the individual stress treatments (*i.e.*, three paired comparisons). Enrichment analysis of GO terms associated with genes identified as differentially expressed (DE) under each individual stress relative to control conditions was then conducted using Fisher’s exact test and GOSeq (Young *et al.* 2010) at a false discovery rate of 0.05. For starvation stress, only genes with a twofold or greater fold change were included in the GO analysis to reduce the dataset to a manageable number. The complete list of GO terms from the analysis of all DE can be found in Table S2. Stress responsive genes common to both heat and starvation were separated into two categories: 1) those up- or down-regulated under both stressors and 2) those regulated in opposing directions (*e.g.*, up-regulated under heat and down-regulated under starvation), and corresponding GO enrichment analyses were performed.

Gene expression levels were validated using RT-qPCR with five genes identified as DE under the various stress conditions: heat shock protein (HSP) 70, acyl-protein thioesterase, cathepsin b-2744, 5′-nucleotidase, and a cuticular protein (CP). Primer pairs were designed using Primer3 (Rozen and Skaletsky 1999) and RT-qPCR was conducted using SYBR Green on the BIO-RAD CFX Connect Real-Time System (Table S3). Ribosomal protein *S9* was used as a reference gene (Bansal *et al.* 2012) and normalized relative expression levels were calculated following methods developed by Hellemans *et al.* (2007) using inter-run calibrators. Three technical replicates were run per biological sample for each gene.

## Results

### Effects of stress on aphid fitness

**Figure 1:**
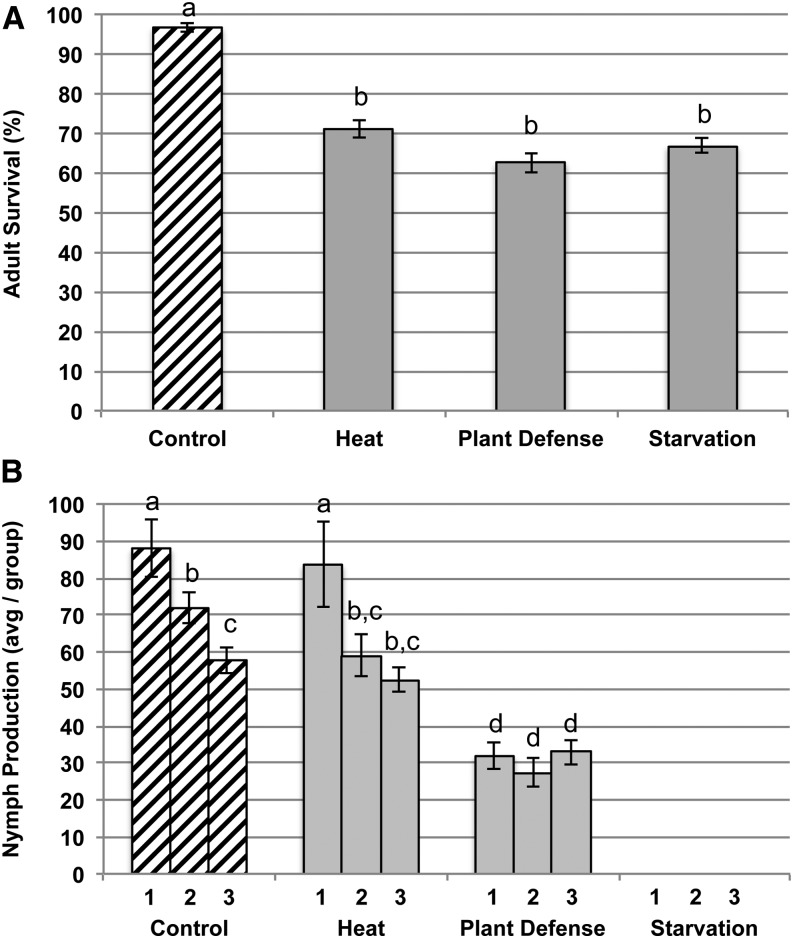
Adult survival and reproduction over 36 hr in *A. glycines* exposed to control conditions and three stressors (plant defense, starvation, and heat). (A) Adult survival averaged across the three experimental blocks. (B) Average nymph production is shown for each separate block due to significant variation across blocks (1, 2, 3) and environments. Letters indicate significant differences in survival and reproductive output (*P* < 0.05).

All three stressors caused on average a 27% reduction in adult survival relative to benign control conditions, which was consistent across experimental blocks (Figure 1A). *Post hoc* tests revealed the three stressors had equivalent survival (*P* values > 0.20) that was significantly lower than under control conditions (*P* values < 0.05). These effects did not vary across the experimental blocks (Environment × Block: F_6,132_ = 0.39, *P* = 0.88). Stress levels with respect to mortality were therefore considered standardized across stress types and experimental blocks. There were significant differences in aphid reproduction under control, heat and plant defensive stress (Environment: F_2,99_ = 43.95, *P* < 0.001) and across experimental blocks (Environment × Block: F_4,99_ = 43.95, *P* < 0.001). Aphids exposed to plant defensive stress produced on average 55% fewer offspring than those under control and heat stressed conditions (*P* values < 0.001) across all blocks, whereas heat stressed and control aphids produced similar numbers of offspring (Figure 1B). Starved aphids did not produce offspring during the 36-hr period.

### Transcriptomic response to stress

**Figure 2:**
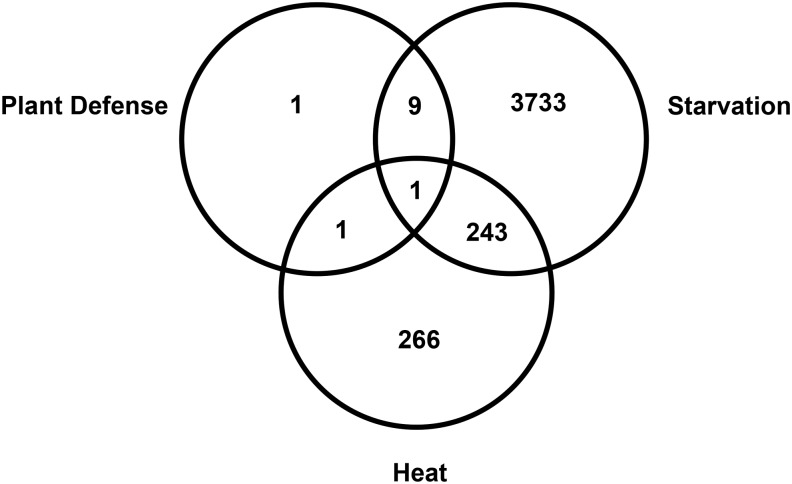
Stress responsive genes in *A. glycines* that are shared and unique to three stressors: plant defense, starvation, and heat. Numbers indicate the combined total of genes significantly up- and down-regulated relative to control conditions.

Overall, transcriptional stress responses were highly variable despite all three stressors, causing an equivalent decrease in adult mortality. The total number of DE genes relative to control conditions differed by several orders of magnitude across the three stress types (Figure 2). Starvation had the strongest effect on gene expression (3985 DE genes), heat stress had an intermediate effect (510 DE genes), and plant defensive stress induced changes in only a handful of genes (12 DE genes). Consequently, there was only one stress responsive gene common to all stressors, a down-regulated 5′ nucleotidase (Figure 2).

Enrichment analysis of stress responsive genes revealed heat stress−induced transcriptional changes involved with general stress response, protein refolding and exoskeletal structure (Table 1). Aphid heat stress response was associated with the up-regulation of HSPs and the down-regulation of CPs. Interestingly, molecular chaperones of both the aphid host and its primary endosymbiont (*Buchnera aphidicola*) showed increased expression. Response to starvation involved increased biosynthesis of basic energy components, nucleosome assembly, and enzymes involved in oxidative processes (Table 1). Starved aphids showed up-regulation of genes involved with glycogen and carbohydrate biosynthesis, histones, cytochrome P450 enzymes, and cysteine proteases. DNA replication genes were down-regulated under starvation, as were genes associated with histone methylation and modification. As with heat stress, there was indication of changes in expression of endosymbiont associated genes under host starvation. Several genes associated with the production of peptidoglycan (murein) involved in bacterial cell wall structure showed increased expression in starved aphids.

**Table 1:** Enrichment of GO terms (FDR < 0.05) and associated stress responsive genes under heat and starvation in *A. glycines*

GO ID	GO Description	Gene Name	Fold Change
**Up-regulated stress responsive pathways and associated genes**			
Heat			
* Response to stress*			
GO:0006950	Response to stress (BP)	ATP-dependent protease	1.7
GO:0006457	Protein folding (BP)	Activator of HSP 90 ATPase	1.4
		HSP 70 (7)	1.5−1.7
		HSP 70 (2)	18.0−24.2
		HSP 40 (DnaJ)*^a^*	1.5
		HSP 70 (DnaK)*^a^* (4)	7.3−7.7
		HSP 60 (GroEL)*^a^* (3)	1.7−1.9
		Stress-induced-phosphoprotein	1.7
		Aldehyde mitochondrial-like	1.6
		Acyl-coa dehydrogenase	1.7
		Caseinolytic peptidase b protein homolog	1.7
		CLN3 battenin	2.7
		Histone partial	2.0
		Ras homolog gene member c	2.2
		Suppressor of g2 allele of skp1 homolog	1.6
		Metastasis suppressor protein 1 (2)	1.5−1.6
Starvation			
* Biosynthesis of basic energy components*			
GO:0000271	Polysaccharide biosynthetic process (BP)	1,4 α-glucan-branching enzyme-like (2)	2.3−2.4
GO:0016051	Carbohydrate biosynthetic process (BP)	6-phosphogluconate decarboxylating-like	2.0
GO:0009250	Glucan biosynthetic process (BP)	α-glucan-branching enzyme-like	2.1
GO:0005978	Glycogen biosynthetic process (BP)	CAMKK-like	2.5
GO:0033692	Cellular polysaccharide biosynthetic process (BP)	Glycogen synthase (2)	2.1
GO:0005976	Polysaccharide metabolic process (BP)	Glycogenin	2.3
		Insulin receptor substrate	2.2
		Phosphoenolpyruvate carboxykinase	2.4
* Biosynthesis of endosymbiont cell wall components*			
GO:0070882	Cellular cell wall organization or biogenesis (BP)	D-alanine-D-alanine ligase*^a^*	3.5
GO:0000270	Peptidoglycan metabolic process (BP)	N-acetylmuramoyl-L-alanine amidase*^a^*(2)	2.4−3.5
		UDP-N-acetylmuramate-alanine ligase*^a^*	6.6
		Peptidoglycan glycosyltransferase*^a^*	6.0
* Nucleosome assembly/organization*			
GO:0034728	Nucleosome assembly (BP)	Histone h3 (5)	2.3−3.4
GO:0000786	Nucleosome organization (BP)	Histone h4	2.4
GO:0006334	Nucleosome (CC)	Histone (3)	2.1−2.6
* Oxidation-reduction/NADP binding*			
GO:0055114	Oxidation-reduction process (MF)	Cytochrome P450 (9)	2.1−5.6
GO:0016491	Oxidoreductase activity (MF)	Fatty acyl-reductase (6)	2.9−7.2
GO:0050661	NADP binding (MF)	Glucose dehydrogenase (3)	2.1−4.9
GO:0003958	NADPH-hemoprotein reductase activity (CC)	Laccase 1	3.6
GO:0009337	Sulfite reductase complex (NADPH) (CC)	Short-chain dehydrogenase reductase	2.1
		3-oxoacyl-acp reductase (2)	4.5−11.4
		NADPH cytochrome P450 reductase (3)	2.8−3.0
		Sulfite reductase β-component (3)	12.9−24.8
* Other processes*			
GO:0008234	Cysteine protease activity (MF)	Cathepsin b (4)	2.0−3.4
		Cathepsin b precursor	2.7
		Cathepsin b-2744 (3)	4.8−5.7
**Down-regulated stress responsive pathways and associated genes**			
Heat			
* Exoskeletal components*			
GO:0042302	Structural constituent of cuticle (MF)	Cuticle protein (2)	1.6−2.1
		Cuticle protein precursor (4)	1.8−3. 5
		RR1 cuticle protein or precursor (3)	1.8
		Endocuticle structural glycoprotein (2)	1.6−1.8
Starvation			
* DNA replication*			
GO:0006260	DNA replication (BP)	DNA polymerase delta catalytic subunit	2.3
GO:0006270	DNA-dependent DNA replication initiation (BP)	DNA primase large subunit (2)	2.3−2.4
GO:0022616	DNA strand elongation (BP)	DNA replication licensing factor (5)	2.0−3.6
		DNA topoisomerase 2-like	2.0
		Flap endonuclease 1	2.8
		gag-pol polyprotein	3.2
		Proliferating cell nuclear antigen (2)	2.5−3.0
		Replication protein (2)	2.0−2.2
		Ribonucleoside-diphosphate reductase large chain	2.9
		rrm1 protein	2.3
		tick partial (2)	2.1
		Uncharacterized transposon-derived protein	3.4
* Histone Methylation and Modification*			
GO:0031061	Negative regulation of histone methylation (BP)	DNA topoisomerase 2-like	2.0
GO:0031057	Negative regulation of histone modification (BP)	DNA (cytosine-5)-methyltransferase 1-like	2.4
GO:0051567	Histone H3-K9 methylation (BP)	Lysine-specific histone demethylase 1	2.3
		DNA primase large subunit	2.3
		Lysine-specific histone	2.0
* Other processes*			
GO:0006002	Fructose 6-phosphate metabolic process (BP)	6-phosphofructokinase (4)	4.2−8.5
			
GO:0000398	mRNA splicing, via spliceosome (BP)	RNA-binding protein cabeza	2.1
		Dead box ATP-dependent RNA helicase	2.4
		Heterogeneous nuclear ribonucleoprotein h2	2.3
			
GO:0051082	Unfolded protein binding (MF)	Calreticulin	2.6
		HSP 40 (DnaJ) homolog (2)	2.0−2.1
		HSP 90 (4)	2.3−3.3
		j domain-containing protein cg6693-like	2
		t-complex protein 1 subunit gamma-like	2.2

Condensed list of GO categories associated with genes up-regulated and down-regulated under stress. Gene lists correspond to each separate GO term and in cases in which genes were associated with multiple terms these GO terms are grouped. The number of gene duplicates is given in parentheses with fold changes relative to control conditions. GO, Gene Ontology; FDR, false discovery rate.

a Indicates genes coded by the aphid primary endosymbiont *Buchnera aphidicola*

In contrast to the strong transcriptional responses elicited by the heat and starvation, no significant enrichment of biological pathways was found under plant defensive stress, likely due to the low overall number of DE genes. Plant defensive stress caused the weakest transcriptional response, with only 12 genes responding relative to control conditions (10 up- and 2 down-regulated), of which only six have a predicted function. Two CPs, acyl-protein thioesterase, a takeout-like protein, and a fatty acid binding protein were up-regulated; whereas a 5′ nucleosidase and gamma-glutamyltranspetidase were down-regulated in response to plant defensive stress.

Although we did not find strong evidence for a core transcriptional defensome across all stressors, there were 242 stress responsive genes common to both heat and starvation stress (Figure 2). Among genes that were regulated in the same direction (188), there was enrichment of *Buchnera* flagellular proteins, cuticular components, and genes associated with protein folding and glycogen biosynthesis (Table 2). Several cuticle proteins and proteins associated with the aphid primary endosymbiont *Buchnera* were down-regulated under heat and starvation. Both aphid host and primary endosymbiont HSP70 were up-regulated under abiotic stress, as were several genes involved in the production of glycogen, including glycogen synthase. For the 54 genes expressed in opposing directions under the two abiotic stressors there was no significant enrichment of associated biological pathways. However, there were several interesting genes with opposing transcriptional responses. Starvation caused a 4- to 7-fold decrease in expression of several cytochrome P450 enzymes, whereas heat increased expression 2- to 3-fold.

**Table 2:** Shared abiotic molecular stress response of *A. glycines*

GO ID	GO Description	Gene Name	Fold Change	
			Heat	Starvation
**Up-Regulated Pathways and Associated Genes**				
GO:0005978	Glycogen biosynthetic process	Glycogen synthase (2)	1.6−1.9	1.7−2.1
		Alpha-glucan-branching enzyme-like	1.5	2.1
		Acyl-coa dehydrogenase	1.7	1.6
GO:0006950	Response to stress	HSP 70 (DnaK) [*Buchnera aphidicola*]	7.3	1.6
		HSP 70 (4)	1.5−1.7	1.4−1.6
		Activator of HSP 90 ATPase	1.4	1.4
		Metastasis suppressor protein 1 (2)	1.5−1.6	1.6−1.8
GO:0003939	L-iditol 2-dehydrogenase activity	Sorbitol dehydrogenase (2)	1.6	2.3
GO:0004794	L-threonine ammonia-lyase activity	Threonine dehydratase deaminase (2)	1.5	2.7
		Map kinase-interacting serine threonine-protein kinase	1.5	3.1
**Down-regulated pathways and associated genes**				
GO:0009424	Bacterial-type flagellum hook	Flagellar hook-associated protein 1 [*Buchnera aphidicola*]	2.6	3.2
GO:0009296	Flagellum assembly	Flagellar protein flgJ [*Buchnera aphidicola*]	2.4	2.6
GO:0001539	Ciliary or flagellar motility	Flagellar basal body P-ring protein [*Buchnera aphidicola*]	3.5	3.0
GO:0042302	Structural constituent of cuticle	Acyl- delta desaturase	2.7	2.5
		Cuticle protein (2)	1.6−1.8	1.5
		GTP cyclohydrolase I	1.5	1.4
		RR1 cuticular protein	1.8	1.4

GO categories (FDR < 0.05) associated with up- and down-regulated genes under heat and starvation stress. The number of gene duplicates is given in parentheses, and fold changes are relative to control conditions. GO, Gene Ontology; FDR, false discovery rate.

Overall, validation of transcriptional levels with RT-qPCR on five stress responsive genes using an independent set of biological samples was consistent with results from RNA-seq analysis. Stress induced fold changes in gene expression estimated using RNA-seq and RT-qPCR were highly correlated (r = 0.96, R^2^ = 0.91) (Figure S1).

## Discussion

Due to the ubiquitous nature of stress, establishing a clear definition and standards of quantification has been challenging. Ambiguity and confusion surrounding the definition of stress has impeded progress toward understanding how organisms cope with environmental challenge from different abiotic and biotic stressors. There is continued debate over the use of a threshold level of intensity to define when an environmental factor is considered stressful (Schulte 2014), and studies often classify a condition as stressful despite minimal, or in some cases no impact on fitness or physiological parameters (Fox and Reed 2011). We chose to standardize stress with respect to survival, an approach few comparative studies have attempted, which enabled us to compare the molecular level effects of different stressors. In the current study, a standardized intensity of stress equal to 27% greater mortality relative to benign conditions resulted in 1–25% of the aphid transcriptome responding (Figures 1 and 2). Not only did the magnitude of response at the molecular level vary tremendously, but stressors induced qualitatively different transcriptional responses with little overlap. Our results highlight the complexity of organismal stress responses and a general need for multilevel approaches to understanding how organisms respond and adapt to variable natural environments.

### Do aphids have a molecular stress defensome*?*

Organisms are known to respond to a variety of abiotic and biotic stressors with the coordinated regulation of a core set of genes and pathways (Kültz 2005; López-Maury *et al.* 2008; Battesti *et al.* 2011). In insects a number of studies have compared transcriptional responses with multiple stressors in targeted sets of genes (*e.g.*, Sinclair *et al.* 2007;Freitak *et al.* 2012; Chen and Zhang 2015); however, few have compared global stress-induced changes to the entire transcriptome (Girardot *et al.* 2004; Sørensen *et al.* 2007; David *et al.* 2010, 2014). Using next-generation sequencing of the soybean aphid transcriptome, we found a single gene (uridine 5′-nucleotidase) DE in response to heat, starvation, and plant defensive stress in the soybean aphid (Figure 2). This lack of support for a core set of transcriptional changes under the three chosen stressors was likely driven by the relatively few responsive genes identified under plant defensive stress (Figure 2). However, among the abiotic stressors examined, there was indication of a common set of transcriptional changes in the soybean aphid (Figure 2), with 188 genes showing similar responses in heat- and starvation-stressed aphids. The most abundant genes in this group were HSPs, which were up-regulated an average of 1.5- to 7-fold relative to control conditions (Table 2). HSPs are a well-established core component of the response to a variety of stressors in many organisms, including insects (Zhao and Jones 2012). In aphids HSPs have also been shown to respond to septic wounding and microbial infection (Altincicek *et al.* 2008), host plant defenses (Francis *et al.* 2010), and insecticides (Silva *et al.* 2012).

Also up-regulated under both abiotic stressors were genes involved in glycogen synthesis (Table 2). Mobilization of energy stores is predicted to mitigate the costs associated with mounting cellular defenses and overall increased metabolic activity often observed under stress (Silva *et al.* 2012). Depletion of energy reserves in the form of glycogen has been demonstrated under temperature, moisture and starvation stress in insects (Storey 1997; Watanabe *et al.* 2002; Gerami 2013). Increased expression of genes involved with the production of glycogen observed in the current study may therefore reflect compensatory mechanisms responding to the depletion of glycogen. Alternatively, increased expression of glycogen synthesis genes may simply represent abnormal transcriptional patterns that could result from direct damage to DNA and/or polypeptides (Wei and Lee 2002) rather than a compensatory or defensive response to stress induced damage or impaired cellular function.

Several CPs showed decreased expression in response to both abiotic stressors (Table 2). The insect cuticle plays an important role in protection against environmental stress (Neven 2000; Benoit 2010), and CPs have been shown to accumulate during exposure to various stresses in insects (Benoit 2010), including aphids (Nguyen *et al.* 2009). In contrast to the down regulation of CPs in response to heat and starvation observed in the present study, recent proteomic work in the potato aphid has shown the accumulation of CPs under thermal stress (Nguyen *et al.* 2009). However, there is a great diversity of CPs in insects, many of which are uncharacterized and their functions unknown (Willis 2010). CPs may therefore be functioning in a stress-responsive role that is unrelated to cuticle formation. The production of certain CPs may also be metabolically costly; therefore, down-regulation could be linked to conservation or redirection of energy reserves under stress.

### Stressor-specific molecular responses

In addition to the large variation in overall magnitude of transcriptional effects across stress types, unique stressor specific responses were also evident (Table 1). Heat-stressed aphids showed up-regulation of genes involved in repair of denatured proteins and down-regulation of exoskeletal components. Heat-induced damage to proteins and corresponding cellular repair mechanisms are well characterized in a broad range of insects (Neven 2000; Zhao and Jones 2012) and enhancement of the cuticular barrier through up-regulation of exoskeletal proteins has been demonstrated under heat stress in aphids (Nguyen *et al.* 2009). The current study is in agreement with previous work showing up-regulation of HSPs in aphids under stress (Nguyen *et al.* 2009); however, we did not find evidence to support heat-induced expression of CPs. This may be because we used adult aphids that had completed their final molt.

Starvation response was associated with increased polysaccharide biosynthesis, expression of histones involved in nucleosome organization, and several cytochrome P450s and cysteine proteases (Table 1). These patterns are in agreement with large-scale metabolic changes associated with mobilization and shifts in allocation of energy reserves observed under starvation stress (Harbison *et al.* 2005; Rion and Kawecki 2007). Starved aphids also showed down-regulation of DNA replication and repair mechanisms, a response that has been documented in a variety of taxa (Kassahn *et al.* 2009; Battesti *et al.* 2011). Decreased functioning of DNA repair mechanisms under stress is hypothesized to enhance mutation rates as an adaptive strategy in bacteria and yeast (Foster 2007; Galhardo *et al.* 2007). Reduced expression of HSP90 also was observed under starvation stress (Table 1), a pattern predicted to generate variation upon which natural selection can act in suboptimal conditions (Jarosz and Lindquist 2010). It is currently unknown whether the molecular mechanisms characterized in other organisms that generate novel genetic variation under stress also are present in aphids.

Exposure to soybean plant defenses associated with the *Rag2* resistance gene elicited the weakest transcriptional response, of which only 6 of 12 genes were functionally annotated. Exposure to soybeans with the *Rag2* gene has been shown to adversely affect the behavior, survival, and reproduction of the soybean aphid (Hill *et al.* 2012; Enders *et al.* 2014). However, the molecular underpinnings of defense pathways associated with soybean resistance (*Rag*) genes and aphid responses are in the initial stages of molecular characterization (Hill *et al.* 2012). Our results suggest *Rag2* may have a targeted effect on aphids, potentially associated with the production of specific allelochemicals. Interestingly, a homolog to the circadian clock regulated protein takeout (to) in *Drosophila* was up-regulated in both starved (eightfold) and *Rag2* (twofold) stressed aphids. In *Drosophila*, takeout is induced under starvation, suggesting this gene may play a role in regulation of energy metabolism and assessment of food availability (Sarov-Blat *et al.* 2000). *Rag2* response to soybean aphid feeding may therefore involve mechanisms aimed at both depriving aphids of nutrients and production of defensive toxins.

Although molecular chaperones are abundantly expressed under stress, different HSP families perform distinct functions and can demonstrate stress specific regulation in insects (Feder and Hofmann 1999; Zhao and Jones 2012; Chen and Zhang 2015). In the soybean aphid we found comparable HSP70 expression levels under both abiotic stressors; however, HSP90 was only DE in starved aphids (Tables 1 and 2). Similarly, Freitak *et al.* (2012) found the magnitude of change in expression of three HSPs in *Tribolium castaneum* varied considerably under heat and starvation stress. Chen and Zhang (2015) have also found that small HSPs respond differently to heat shock, starvation and oxidative stress in the diamondback moth (*Plutella xylostella*).

### Aphid endosymbiont stress response

There is growing evidence that symbiotic relationships play a prominent role in host adaptation to environmental stress (Feldhaar 2011; Gilbert *et al.* 2010). Insect endosymbionts have been shown to influence parasite and pathogen resistance, heat tolerance, and even manipulate interactions between insects and their host plants (see reviews in Feldhaar 2011 and Frago *et al.* 2012). However, host-symbiont dynamics under stress remain poorly understood.

Several studies indicate the transcriptome of the primary aphid endosymbiont, *Buchnera aphidicola*, may be relatively stable under various environmental stressors (Baumann *et al.* 1996; Moran and Degnan 2006; Nguyen *et al.* 2007). However, recent proteomic work in the potato aphid indicated differences in the stress-responsive accumulation of different isoforms of the *B. aphidicola* chaperone protein GroEL (Nguyen *et al.* 2009). In the current study multiple *B. aphidicola* HSPs were up-regulated under heat and starvation stress (Table 1). Recent work by Poliakov *et al.* (2011) confirms that molecular chaperones such as GroEL and DnaK (HSP70) are dominant features of the *Buchnera* proteome, further suggesting their central role in the protection and maintenance of an endosymbiotic lifestyle (Fares *et al.* 2004).

Several genes involved in metabolism of peptidoglycan, a component of the bacterial cell wall, were up-regulated in starved aphids (Table 1), which could be a defensive response or indicative of bacterial turnover in stressed endosymbiont populations. Endosymbiont flagellar hook and basal body transcripts were also down-regulated under heat and starvation stress (Table 2). Genes involved in bacteria flagellular assembly are often lost or undergo functional changes in endosymbionts as a result of adapting to a nonmotile intracellular lifestyle (Toft and Fares 2008). Although the exact function of these flagellar genes is unknown in *Buchner*a, it has been suggested they may play a role in protein transport between bacterium and host (Maezawa *et al.* 2006). Decreased expression of flagellar genes may either reflect impaired functioning of *Buchnera* under stress or an overall reduction in bacterial titer levels.

## Future directions

Adaptation to complex stressful environments likely involves fine-tuned responses as well as a flexible core defense response to a diversity of adverse conditions (Mittler 2006; Sørensen *et al.* 2007; Atkinson and Urwin 2012). Work in *Drosophila* has shown that both stressor-specific and general molecular responses are present in populations selected for tolerance to a variety of abiotic stressors such as heat, starvation, and desiccation (Sørensen *et al.* 2007). However, the overall magnitude of molecular response may vary across multicellular organisms, with some exhibiting weak or no global stress response systems (López-Maury *et al.* 2008). Results from the current study, as well as work in *Aedes* (David *et al.* 2010) and *Drosophila* (Girardot *et al.* 2004; Sørensen *et al.* 2007), suggest core insect responses to abiotic stressors may be comprised of several hundred genes or less. Variation in a number of factors, including stress level and insect life history stage, likely influence detection of global patterns consistent with the stress defensome hypothesis. Fold changes of stress responsive genes also should be considered in comparative studies. Stress-induced fold changes in the soybean aphid ranged from ~1.5 to greater than 20; however, in some cases 100-fold and greater up and down-regulation of genes has been observed under multiple stressors in insects (Sinclair *et al.* 2007; David *et al.* 2010).

In the current study, stressor-specific transcriptional responses were prevalent and there was minimal overlap across the different stressors. However, further research exploring response to additional stressors, both alone and in combination, is needed to fully understand the adaptive role of the core aphid defensome *vs.* stressor-specific responses. For example, aspects of the shared transcriptional response to heat and starvation also may be present under other stressors, such as those mediated via the host plant or resulting from exposure to xenobiotics. It is also unknown whether fundamental differences exist between abiotic and biotic stress responses. A distinguishing feature of biotic stress interactions is the potential for coevolution to occur, particularly in plant-insect systems. Identifying similarities and conflicts in response to abiotic and biotic forms of stress could begin to illuminate the molecular basis of long-term adaptive processes in multistress environments.

Incorporation of additional aphid genotypes also is needed to further our understanding of the ecological and adaptive relevance of the transcriptional stress responses observed in this study. Significant genotype by stress interactions have been demonstrated in aphids for survival, reproduction, and behavior (Ferrari *et al.* 2001; Cardoza *et al.* 2006; Lombaert *et al.* 2009; Enders *et al.* 2014). Important differences at the molecular level could potentially underlie intraspecific variation observed under stress at the fitness level. Research exploring the extent to which transcriptional responses are stable across different aphid genotypes will aid in determining the role of a core molecular defensome *vs.* stressor-specific responses.

Although stress level was equivalent with respect to adult mortality, we found the impact of the different stressors on offspring production varied significantly (Figure 1). Response to stress involves balancing potentially conflicting demands between growth, reproduction, and long-term survival (López-Maury *et al.* 2008; Atkinson and Urwin 2012), which could contribute to differences across fitness components. During brief periods of starvation, aphids have been shown to delay reproduction (Kouamé and Mackauer 1992; Xu *et al.* 2012) and in some cases selective reabsorption of embryos is known to occur (Ward and Dixon 1982; Stadler 1995). When deprived of food, aphids potentially allocate resources to maintenance of basic biological functions first and then to reproduction (Kouamé and Mackauer 1992), which is in line with our results showing a lack of reproduction in starved aphids. In contrast, heat stress did not affect reproductive output, which may be explained by generally faster insect development at greater temperatures (Campbell *et al.* 1974) offsetting the loss of nymphs due to stress induced mortality. Finally, although the exact defensive mechanisms affecting aphid survival and reproduction on *Rag2* soybean are unknown, similar reductions in both fitness measures have been previously reported (Enders *et al.* 2014).

Overall, our results demonstrate that equivalent levels of stress imposed on aphid survival can have profoundly different effects on molecular level responses and components of fitness. One explanation for the observed transcriptional differences across stress types is that equivalent mortality may be achieved through different mechanisms for each stressor. Variation in the regulation of gene expression affecting the speed of induction and duration of response could also contribute to transcriptional differences across stress types (De Nadal *et al.* 2011). For example, stress inflicted by *Rag2* soybean defenses may elicit strong but transient transcriptional changes earlier than 36 hr, potentially explaining why few genes were found DE. Alternatively, impaired ability of aphid sensory systems to detect a particular form of plant defensive stress (*e.g.*, allelochemical) could result in delayed or minimal induction of cellular stress response pathways. Overall, our results suggest the magnitude of stress applied, timing of measurement and variation across fitness components should be considered when interpreting results from comparative multilevel stress studies.

## Supplementary Material

Supporting Information
