# Supplementary material for: Abiotic and Biotic Stressors Causing Equivalent Mortality Induce Highly Variable Transcriptional Responses in the Soybean Aphid
Source: G3 (Bethesda). 2014 Dec 23;5(2):261–70. doi: 10.1534/g3.114.015149 (PMC4321034; doi:10.1534/g3.114.015149)
Supplement: Supporting Information [file supp_5_2_261__index.html]

Abiotic and Biotic Stressors Causing Equivalent Mortality Induce Highly Variable Transcriptional Responses in the Soybean Aphid — Supporting Information 

# Abiotic and Biotic Stressors Causing Equivalent Mortality Induce Highly Variable Transcriptional Responses in the Soybean Aphid

## Supporting Information for Enders *et al.*, 2015

**Files in this Data Supplement:**

- Supporting Information - Tables S1-S3 and Figure S1 (PDF, 209 KB)
- Table S1 - Aphid fitness data under control and stressful conditions. (PDF, 144 KB)
- Table S2 - Enrichment analysis of stress responsive genes in *A. glycines*. (PDF, 163 KB)
- Table S3 - Primer information for 5 stress responsive genes and a reference gene (*RPS9*) used for RNAseq validation using RT-qPCR. (PDF, 147 KB)
- Figure S1 - Linear regression of stress induced fold changes in 5 genes using RNAseq and qRT-PCR. (PDF, 139 KB)
