# Supplementary material for: Abiotic and Biotic Stressors Causing Equivalent Mortality Induce Highly Variable Transcriptional Responses in the Soybean Aphid
Source: G3 (Bethesda). 2014 Dec 23;5(2):261–70. doi: 10.1534/g3.114.015149 (PMC4321034; doi:10.1534/g3.114.015149)
Supplement: Supporting Information [file supp_g3.114.015149_015149SI.pdf]

G3·Genes | Genomes | Genetics

Abiotic and biotic stressors causing equivalent mortality induce highly variable transcriptional responses in the Soybean Aphid

Laramy S. Enders[*], Ryan D. Bickel[§,†], Jennifer A. Brisson[§,†], Tiffany M. Heng-Moss[*], Blair D. Siegfried[*], Anthony J. Zera[§] and Nicholas J. Miller[*]

[*] Department of Entomology, University of Nebraska-Lincoln, Lincoln, NE USA 68583-0816,
[§] School of Biological Sciences, University of Nebraska-Lincoln, Lincoln, NE USA 68583-0118
[†] Department of Biology, University of Rochester, NY USA 14627-0211

Corresponding Author: Nicholas J. Miller, Department of Entomology, University of Nebraska-Lincoln, Lincoln, NE USA 68583-0816

DOI: 10.1534/g3.114.015149

Table S1 Aphid fitness data under control and stressful conditions. Aphid survival (# adults) and reproduction (# Nymphs) was recorded after 36 h exposure to four different environments (ENV: control, heat, starvation, and RAG2 plant defensive stress). Twelve replicate cages were recorded for each environmental treatment in 3 separate experimental Blocks.

ENV	Replicate	# Adults	# Nymphs	Block
CON	1	20	81	1
CON	2	20	128	1
CON	3	20	96	1
CON	4	20	122	1
CON	5	20	92	1
CON	6	19	53	1
CON	7	20	52	1
CON	8	19	80	1
CON	9	14	62	1
CON	10	20	132	1
CON	11	18	81	1
CON	12	20	77	1
CON	1	20	81	2
CON	2	20	65	2
CON	3	19	78	2
CON	4	20	84	2
CON	5	20	95	2
CON	6	20	91	2
CON	7	19	76	2
CON	8	17	54	2
CON	9	16	47	2
CON	10	20	68	2
CON	11	20	63	2
CON	12	20	60	2
HEAT	1	11	69	1
HEAT	2	15	85	1
HEAT	3	17	127	1
HEAT	4	19	114	1
HEAT	5	13	115	1
HEAT	6	19	97	1
HEAT	7	17	120	1
HEAT	8	12	100	1

HEAT	9	15	14	1
HEAT	10	14	79	1
HEAT	11	18	80	1
HEAT	12	11	3	1
HEAT	1	14	62	2
HEAT	2	15	52	2
HEAT	3	13	32	2
HEAT	4	13	40	2
HEAT	5	13	68	2
HEAT	6	14	24	2
HEAT	7	10	88	2
HEAT	8	15	62	2
HEAT	9	18	80	2
HEAT	10	18	74	2
HEAT	11	11	76	2
HEAT	12	15	51	2
STARVE	1	8	0	1
STARVE	2	16	0	1
STARVE	3	15	0	1
STARVE	4	11	0	1
STARVE	5	16	0	1
STARVE	6	16	0	1
STARVE	7	13	0	1
STARVE	8	15	0	1
STARVE	9	14	0	1
STARVE	10	13	0	1
STARVE	11	14	0	1
STARVE	12	15	0	1
STARVE	1	15	0	2
STARVE	2	15	0	2
STARVE	3	9	0	2
STARVE	4	14	0	2
STARVE	5	15	0	2
STARVE	6	12	0	2
STARVE	7	14	0	2
STARVE	8	14	0	2
STARVE	9	13	0	2
STARVE	10	13	0	2
STARVE	11	12	0	2

STARVE	12	14	0	2
RAG2	1	14	28	1
RAG2	2	14	25	1
RAG2	3	16	20	1
RAG2	4	16	14	1
RAG2	5	16	46	1
RAG2	6	12	29	1
RAG2	7	12	21	1
RAG2	8	12	24	1
RAG2	9	14	34	1
RAG2	10	16	52	1
RAG2	11	7	51	1
RAG2	12	10	38	1
RAG2	1	14	36	2
RAG2	2	19	57	2
RAG2	3	10	39	2
RAG2	4	16	40	2
RAG2	5	13	12	2
RAG2	6	10	20	2
RAG2	7	5	12	2
RAG2	8	13	24	2
RAG2	9	12	28	2
RAG2	10	14	22	2
RAG2	11	16	23	2
RAG2	12	10	16	2
CON	1	19	57	3
CON	2	20	70	3
CON	3	19	61	3
CON	4	19	73	3
CON	5	20	45	3
CON	6	20	51	3
CON	7	19	31	3
CON	8	19	56	3
CON	9	20	74	3
CON	10	20	56	3
CON	11	20	59	3
CON	12	20	60	3
HEAT	1	16	66	3
HEAT	2	17	73	3

HEAT	3	15	49	3
HEAT	4	12	51	3
HEAT	5	12	48	3
HEAT	6	12	44	3
HEAT	7	12	54	3
HEAT	8	13	64	3
HEAT	9	13	56	3
HEAT	10	11	31	3
HEAT	11	15	48	3
HEAT	12	13	45	3
STARVE	1	13	0	3
STARVE	2	16	0	3
STARVE	3	10	0	3
STARVE	4	16	0	3
STARVE	5	13	0	3
STARVE	6	13	0	3
STARVE	7	10	0	3
STARVE	8	10	0	3
STARVE	9	15	0	3
STARVE	10	14	0	3
STARVE	11	13	0	3
STARVE	12	12	0	3
RAG2	1	14	17	3
RAG2	2	9	21	3
RAG2	3	14	56	3
RAG2	4	9	34	3
RAG2	5	12	27	3
RAG2	6	11	39	3
RAG2	7	10	17	3
RAG2	8	13	34	3
RAG2	9	11	39	3
RAG2	10	8	37	3
RAG2	11	17	45	3
RAG2	12	12	29	3

Table S2 Enrichment analysis of stress responsive genes in *A. glycines*. GO terms associated with genes up- and down- regulated under heat and starvation stress (FDR < 0.05).

GO ID	GO Description	# Contigs
HEAT UP-REGULATED		
GO:0006950	response to stress (BP)	26
GO:0006457	protein folding (BP)	12
HEAT DOWN-REGULATED		
GO:0042302	structural constituent of cuticle (MF)	11
STARVATION UP-REGULATED		
GO:0003824	catalytic activity (MF)	180
GO:0016491	oxidoreductase activity (MF)	43
GO:0055114	oxidation-reduction process (MF)	40
GO:0048037	cofactor binding (MF)	23
GO:0050662	coenzyme binding (MF)	16
GO:0005506	iron ion binding (MF)	15
GO:0005976	polysaccharide metabolic process (BP)	14
GO:0016051	carbohydrate biosynthetic process (BP)	13
GO:0000271	polysaccharide biosynthetic process (BP)	11
GO:0034728	nucleosome assembly (BP)	8
GO:0000786	nucleosome organization (BP)	8
GO:0006334	nucleosome (CC)	8
GO:0008234	cysteine protease activity (MF)	8
GO:0065004	protein-DNA complex assembly (BP)	8
GO:0071824	protein-DNA complex subunit organization (BP)	8
GO:0006333	chromatin assembly or disassembly (BP)	8
GO:0034637	cellular carbohydrate biosynthetic process (BP)	8
GO:0033692	cellular polysaccharide biosynthetic process (BP)	7
GO:0050661	NADP binding (MF)	7
GO:0034637	cellular carbohydrate biosynthetic process	7
GO:0009250	glucan biosynthetic process (BP)	6
GO:0005978	glycogen biosynthetic process (BP)	6
GO:0000270	peptidoglycan metabolic process (BP)	6
GO:0071555	cell wall organization (BP)	6
GO:0007047	cellular cell wall organization (BP)	6
GO:0070882	cellular cell wall organization or biogenesis (BP)	6
GO:0071554	cell wall organization or biogenesis (BP)	6
GO:0003958	NADPH-hemoprotein reductase activity (CC)	3
GO:0009337	sulfite reductase complex (NADPH) (CC)	3
GO:0016653	NADPH, heme protein as acceptor (MF)	3

GO:0003844	1,4-alpha-glucan branching enzyme activity (MF)	3
GO:0004783	sulfite reductase (NADPH) activity (MF)	3
STARVATION DOWN-REGULATED		
GO:0006260	DNA replication (BP)	15
GO:0006261	DNA-dependent DNA replication (BP)	12
GO:0019843	rRNA binding (MF)	11
GO:0051082	unfolded protein binding (MF)	9
GO:0006270	DNA-dependent DNA replication initiation (BP)	6
GO:0006271	DNA strand elongation involved in DNA replication (BP)	5
GO:0022616	DNA strand elongation (BP)	5
GO:0051567	histone H3-K9 methylation (BP)	4
GO:0008443	phosphofructokinase activity (BP)	4
GO:0006002	fructose 6-phosphate metabolic process (BP)	4
GO:0006312	mitotic recombination (BP)	4
GO:0005945	6-phosphofructokinase complex (CC)	4
GO:0008443	phosphofructokinase activity (MF)	4
GO:0051570	regulation of histone H3-K9 methylation (BP)	3
GO:0031061	negative regulation of histone methylation (BP)	3
GO:0031057	negative regulation of histone modification (BP)	3
GO:0000398	mRNA splicing, via spliceosome (BP)	3

Table S3 Primer information for 5 stress responsive genes and a reference gene (*RPS9*) used for RNAseq validation using RT-qPCR.

Gene Name	Primer	Primer Sequence (5'-3')	Amplicon (bp)	Primer Efficiency (%)	R^2
acyl-protein thioesterase	For	AGGCAGATGACTTTGACGTT	104	98.5	0.99
	Rev	CACACAGGCACCATCATATT			
cathepsin b-2744	For	GGAATCGAAGTTACCACCAG	122	91.7	0.99
	Rev	CCGCTCAAAACCTAATGTCT			
heat shock protein 70 (*HSP70*)	For	ATTGTTGTCCAACCACTGGA	106	71.5	0.98
	Rev	CAGTGTTAAACAAGCGTTGG			
cuticular protein rr-1 motif 32	For	TCGTTAGACAATCGCAAGAA	120	119.6	0.99
	Rev	GAACAGCGTTATCAGTTCCA			
5'-nucleotidase (*5-ecto*)	For	TTGGCAAATGGTGGTAATAA	111	103.5	0.94
	Rev	GGGACTTATGGATTGCATGT			
40S ribosomal protein S9 (*RPS9*)	For	ACAGATTAAGAGGAACGATTA	95	75.5	0.97
	Rev	GGAAGAACTTGAGGAAGG			

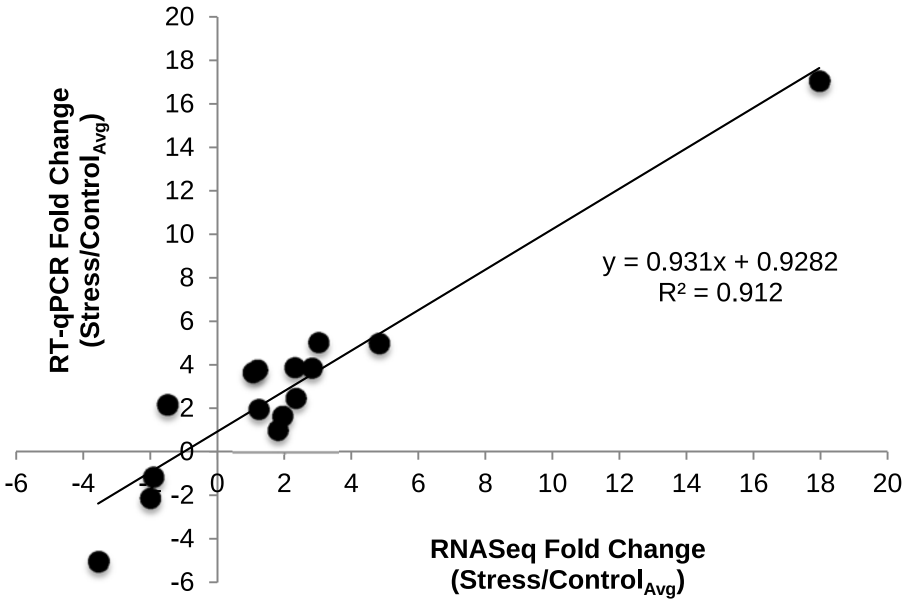

Figure S1 Linear regression of stress induced fold changes in 5 genes using RNAseq and qRT-PCR. For each gene the fold changes for each stressor relative to the average of the control replicates were calculated using either number of raw reads (RNAseq) or CNRQ values (qRT-PCR: see Hellemans et al. 2007).
