# Supplementary material for: Abiotic and Biotic Stressors Causing Equivalent Mortality Induce Highly Variable Transcriptional Responses in the Soybean Aphid
Source: G3 (Bethesda). 2014 Dec 23;5(2):261–70. doi: 10.1534/g3.114.015149 (PMC4321034; doi:10.1534/g3.114.015149)
Supplement: Supporting Information [file supp_g3.114.015149_TableS1.pdf]

Table S1 Aphid fitness data under control and stressful conditions. Aphid survival (# adults) and reproduction (# Nymphs) was recorded after 36 h exposure to four different environments (ENV: control, heat, starvation, and RAG2 plant defensive stress). Twelve replicate cages were recorded for each environmental treatment in 3 separate experimental Blocks.

ENV	Replicate	# Adults	# Nymphs	Block
CON	1	20	81	1
CON	2	20	128	1
CON	3	20	96	1
CON	4	20	122	1
CON	5	20	92	1
CON	6	19	53	1
CON	7	20	52	1
CON	8	19	80	1
CON	9	14	62	1
CON	10	20	132	1
CON	11	18	81	1
CON	12	20	77	1
CON	1	20	81	2
CON	2	20	65	2
CON	3	19	78	2
CON	4	20	84	2
CON	5	20	95	2
CON	6	20	91	2
CON	7	19	76	2
CON	8	17	54	2
CON	9	16	47	2
CON	10	20	68	2
CON	11	20	63	2
CON	12	20	60	2
HEAT	1	11	69	1
HEAT	2	15	85	1
HEAT	3	17	127	1
HEAT	4	19	114	1
HEAT	5	13	115	1
HEAT	6	19	97	1
HEAT	7	17	120	1
HEAT	8	12	100	1

HEAT	9	15	14	1
HEAT	10	14	79	1
HEAT	11	18	80	1
HEAT	12	11	3	1
HEAT	1	14	62	2
HEAT	2	15	52	2
HEAT	3	13	32	2
HEAT	4	13	40	2
HEAT	5	13	68	2
HEAT	6	14	24	2
HEAT	7	10	88	2
HEAT	8	15	62	2
HEAT	9	18	80	2
HEAT	10	18	74	2
HEAT	11	11	76	2
HEAT	12	15	51	2
STARVE	1	8	0	1
STARVE	2	16	0	1
STARVE	3	15	0	1
STARVE	4	11	0	1
STARVE	5	16	0	1
STARVE	6	16	0	1
STARVE	7	13	0	1
STARVE	8	15	0	1
STARVE	9	14	0	1
STARVE	10	13	0	1
STARVE	11	14	0	1
STARVE	12	15	0	1
STARVE	1	15	0	2
STARVE	2	15	0	2
STARVE	3	9	0	2
STARVE	4	14	0	2
STARVE	5	15	0	2
STARVE	6	12	0	2
STARVE	7	14	0	2
STARVE	8	14	0	2
STARVE	9	13	0	2
STARVE	10	13	0	2
STARVE	11	12	0	2

STARVE	12	14	0	2
RAG2	1	14	28	1
RAG2	2	14	25	1
RAG2	3	16	20	1
RAG2	4	16	14	1
RAG2	5	16	46	1
RAG2	6	12	29	1
RAG2	7	12	21	1
RAG2	8	12	24	1
RAG2	9	14	34	1
RAG2	10	16	52	1
RAG2	11	7	51	1
RAG2	12	10	38	1
RAG2	1	14	36	2
RAG2	2	19	57	2
RAG2	3	10	39	2
RAG2	4	16	40	2
RAG2	5	13	12	2
RAG2	6	10	20	2
RAG2	7	5	12	2
RAG2	8	13	24	2
RAG2	9	12	28	2
RAG2	10	14	22	2
RAG2	11	16	23	2
RAG2	12	10	16	2
CON	1	19	57	3
CON	2	20	70	3
CON	3	19	61	3
CON	4	19	73	3
CON	5	20	45	3
CON	6	20	51	3
CON	7	19	31	3
CON	8	19	56	3
CON	9	20	74	3
CON	10	20	56	3
CON	11	20	59	3
CON	12	20	60	3
HEAT	1	16	66	3
HEAT	2	17	73	3

HEAT	3	15	49	3
HEAT	4	12	51	3
HEAT	5	12	48	3
HEAT	6	12	44	3
HEAT	7	12	54	3
HEAT	8	13	64	3
HEAT	9	13	56	3
HEAT	10	11	31	3
HEAT	11	15	48	3
HEAT	12	13	45	3
STARVE	1	13	0	3
STARVE	2	16	0	3
STARVE	3	10	0	3
STARVE	4	16	0	3
STARVE	5	13	0	3
STARVE	6	13	0	3
STARVE	7	10	0	3
STARVE	8	10	0	3
STARVE	9	15	0	3
STARVE	10	14	0	3
STARVE	11	13	0	3
STARVE	12	12	0	3
RAG2	1	14	17	3
RAG2	2	9	21	3
RAG2	3	14	56	3
RAG2	4	9	34	3
RAG2	5	12	27	3
RAG2	6	11	39	3
RAG2	7	10	17	3
RAG2	8	13	34	3
RAG2	9	11	39	3
RAG2	10	8	37	3
RAG2	11	17	45	3
RAG2	12	12	29	3
