# Supplementary material for: Abiotic and Biotic Stressors Causing Equivalent Mortality Induce Highly Variable Transcriptional Responses in the Soybean Aphid
Source: G3 (Bethesda). 2014 Dec 23;5(2):261–70. doi: 10.1534/g3.114.015149 (PMC4321034; doi:10.1534/g3.114.015149)
Supplement: Supporting Information [file supp_g3.114.015149_TableS2.pdf]

Table S2 Enrichment analysis of stress responsive genes in *A. glycines*. GO terms associated with genes up- and down- regulated under heat and starvation stress (FDR < 0.05).

GO ID	GO Description	# Contigs
HEAT UP-REGULATED		
GO:0006950	response to stress (BP)	26
GO:0006457	protein folding (BP)	12
HEAT DOWN-REGULATED		
GO:0042302	structural constituent of cuticle (MF)	11
STARVATION UP-REGULATED		
GO:0003824	catalytic activity (MF)	180
GO:0016491	oxidoreductase activity (MF)	43
GO:0055114	oxidation-reduction process (MF)	40
GO:0048037	cofactor binding (MF)	23
GO:0050662	coenzyme binding (MF)	16
GO:0005506	iron ion binding (MF)	15
GO:0005976	polysaccharide metabolic process (BP)	14
GO:0016051	carbohydrate biosynthetic process (BP)	13
GO:0000271	polysaccharide biosynthetic process (BP)	11
GO:0034728	nucleosome assembly (BP)	8
GO:0000786	nucleosome organization (BP)	8
GO:0006334	nucleosome (CC)	8
GO:0008234	cysteine protease activity (MF)	8
GO:0065004	protein-DNA complex assembly (BP)	8
GO:0071824	protein-DNA complex subunit organization (BP)	8
GO:0006333	chromatin assembly or disassembly (BP)	8
GO:0034637	cellular carbohydrate biosynthetic process (BP)	8
GO:0033692	cellular polysaccharide biosynthetic process (BP)	7
GO:0050661	NADP binding (MF)	7
GO:0034637	cellular carbohydrate biosynthetic process	7
GO:0009250	glucan biosynthetic process (BP)	6
GO:0005978	glycogen biosynthetic process (BP)	6
GO:0000270	peptidoglycan metabolic process (BP)	6
GO:0071555	cell wall organization (BP)	6
GO:0007047	cellular cell wall organization (BP)	6
GO:0070882	cellular cell wall organization or biogenesis (BP)	6
GO:0071554	cell wall organization or biogenesis (BP)	6
GO:0003958	NADPH-hemoprotein reductase activity (CC)	3
GO:0009337	sulfite reductase complex (NADPH) (CC)	3
GO:0016653	NADPH, heme protein as acceptor (MF)	3

GO:0003844	1,4-alpha-glucan branching enzyme activity (MF)	3
GO:0004783	sulfite reductase (NADPH) activity (MF)	3
STARVATION DOWN-REGULATED		
GO:0006260	DNA replication (BP)	15
GO:0006261	DNA-dependent DNA replication (BP)	12
GO:0019843	rRNA binding (MF)	11
GO:0051082	unfolded protein binding (MF)	9
GO:0006270	DNA-dependent DNA replication initiation (BP)	6
GO:0006271	DNA strand elongation involved in DNA replication (BP)	5
GO:0022616	DNA strand elongation (BP)	5
GO:0051567	histone H3-K9 methylation (BP)	4
GO:0008443	phosphofructokinase activity (BP)	4
GO:0006002	fructose 6-phosphate metabolic process (BP)	4
GO:0006312	mitotic recombination (BP)	4
GO:0005945	6-phosphofructokinase complex (CC)	4
GO:0008443	phosphofructokinase activity (MF)	4
GO:0051570	regulation of histone H3-K9 methylation (BP)	3
GO:0031061	negative regulation of histone methylation (BP)	3
GO:0031057	negative regulation of histone modification (BP)	3
GO:0000398	mRNA splicing, via spliceosome (BP)	3
