# Supplementary material for: Abiotic and Biotic Stressors Causing Equivalent Mortality Induce Highly Variable Transcriptional Responses in the Soybean Aphid
Source: G3 (Bethesda). 2014 Dec 23;5(2):261–70. doi: 10.1534/g3.114.015149 (PMC4321034; doi:10.1534/g3.114.015149)
Supplement: Supporting Information [file supp_g3.114.015149_TableS3.pdf]

Table S3 Primer information for 5 stress responsive genes and a reference gene (*RPS9*) used for RNAseq validation using RT-qPCR.

Gene Name	Primer	Primer Sequence (5'-3')	Amplicon (bp)	Primer Efficiency (%)	R^2
acyl-protein thioesterase	For	AGGCAGATGACTTTGACGTT	104	98.5	0.99
	Rev	CACACAGGCACCATCATATT			
cathepsin b-2744	For	GGAATCGAAGTTACCACCAG	122	91.7	0.99
	Rev	CCGCTCAAAACCTAATGTCT			
heat shock protein 70 (*HSP70*)	For	ATTGTTGTCCAACCACTGGA	106	71.5	0.98
	Rev	CAGTGTTAAACAAGCGTTGG			
cuticular protein rr-1 motif 32	For	TCGTTAGACAATCGCAAGAA	120	119.6	0.99
	Rev	GAACAGCGTTATCAGTTCCA			
5'-nucleotidase (*5-ecto*)	For	TTGGCAAATGGTGGTAATAA	111	103.5	0.94
	Rev	GGGACTTATGGATTGCATGT			
40S ribosomal protein S9 (*RPS9*)	For	ACAGATTAAGAGGAACGATTA	95	75.5	0.97
	Rev	GGAAGAACTTGAGGAAGG			
