# Supplementary material for: Abiotic and Biotic Stressors Causing Equivalent Mortality Induce Highly Variable Transcriptional Responses in the Soybean Aphid
Source: G3 (Bethesda). 2014 Dec 23;5(2):261–70. doi: 10.1534/g3.114.015149 (PMC4321034; doi:10.1534/g3.114.015149)
Supplement: Supporting Information [file supp_g3.114.015149_FigureS1.pdf]

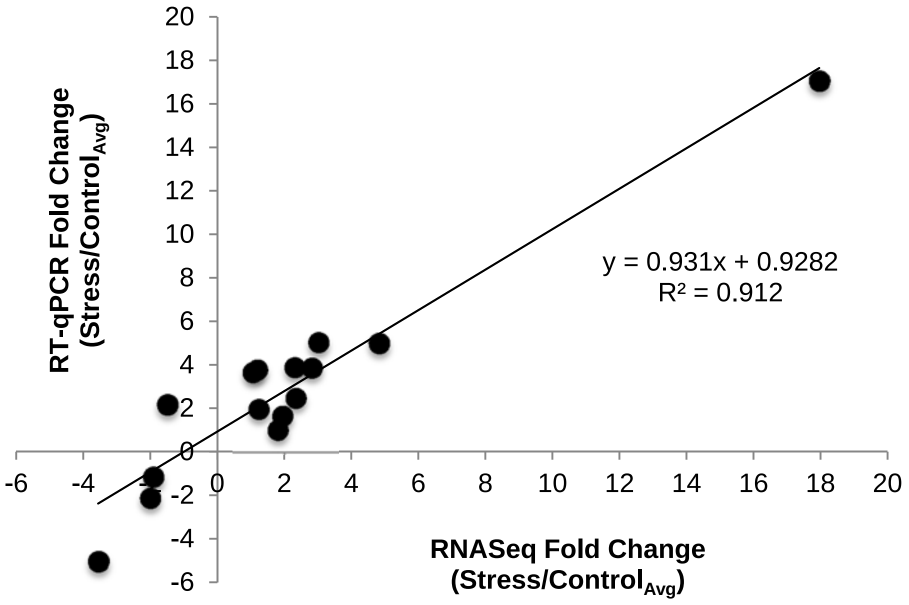

Figure S1 Linear regression of stress induced fold changes in 5 genes using RNAseq and qRT-PCR. For each gene the fold changes for each stressor relative to the average of the control replicates were calculated using either number of raw reads (RNAseq) or CNRQ values (qRT-PCR: see Hellemans et al. 2007).
